# Piezoelectric, Mechanical and Acoustic Properties of KNaNbOF_5_ from First-Principles Calculations

**DOI:** 10.3390/ma8125477

**Published:** 2015-12-09

**Authors:** Han Han, Cheng Cheng, Xiao-Gen Xiong, Jing Su, Jian-Xing Dai, Hui Wang, Gen Yin, Ping Huai

**Affiliations:** 1 Shanghai Institute of Applied Physics, Chinese Academy of Sciences, Shanghai 201800, China; chengcheng@sinap.ac.cn (C.C.); xiongxiaogen@sinap.ac.cn (X.-G.X.); sujing@sinap.ac.cn (J.S.); daijianxing@sinap.ac.cn (J.-X.D.); 2 School of Physics and Engineering, Henan University of Science and Technology, Luoyang 471003, China; nkxirainbow@gmail.com; 3 Department of Electrical Engineering, University of California, Riverside, CA 92521-0204, USA; gen.yin@email.ucr.edu

**Keywords:** piezoelectric materials, density functional calculation, mechanical properties, acoustic properties

## Abstract

Recently, a noncentrosymmetric crystal, KNaNbOF_5_, has attracted attention due to its potential to present piezoelectric properties. Although α- and β-KNaNbOF_5_ are similar in their stoichiometries, their structural frameworks, and their synthetic routes, the two phases exhibit very different properties. This paper presents, from first-principles calculations, comparative studies of the structural, electronic, piezoelectric, and elastic properties of the α and the β phase of the material. Based on the Christoffel equation, the slowness surface of the acoustic waves is obtained to describe its acoustic prosperities. These results may benefit further applications of KNaNbOF_5_.

## 1. Introduction

Global innovations in electronic devices are driving the demand for new piezoelectric materials with improved properties. These materials are of great importance for a variety of technological applications including the production and detection of sound, the generation of high voltages, positioning in scanning probe microscopes, and the implementation of second harmonic generation (SHG) devices [1]. Moreover, piezoelectric materials are also known as the “smart” components in radiation environments, especially in the nuclear industry. During the past several years, piezoelectric technology has been revolutionizing sensing in reactor and waste vessel environments for the control and safety protection of nuclear reactors [2,3]. Piezoelectric sensing is important to the measurement of many control variables, such as temperature, pressure, flow, and neutron flux. The importance of piezoelectricity in these areas demands the search for novel piezoelectric materials of different varieties with better performances.

A noncentrosymmetric crystal, KNaNbOF_5_, has been proposed as a candidate piezoelectric material. It has exhibited many interesting and intriguing properties. Firstly, oxy-fluoride perovskite is not a common piezoelectric structure [4] compared to the well-known perovskite materials with the general formula ABO_3_ [5,6,7,8]. The piezoelectric mechanism in KNaNbOF_5_ is very special. The oxygen and fluorine atoms can co-exist in one framework, [NbOF_5_]^2−^. In this framework, each Nb is surrounded by five fluorine anions and one oxygen anion. These atoms form a distorted octahedron, where the distortion is caused by the different bond lengths to the centric Nb^5+^ ion. Thus, a net dipole is generated by the Nb^5+^ ion moving from the center of the O-F cage to the oxygen side. Secondly, KNaNbOF_5_ is also a triboluminescent material. It was reported that strong triboluminescence can be visible to the naked eye under normal lighting conditions [9]. Last but not least, KNaNbOF_5_ has attracted great interest due to its polymorphism [10,11]. For the same identities of the cations and anions, KNaNbOF_5_ has two different phases that exhibit very different behaviors. One phase, the α-KNaNbOF_5,_ is a polar noncentrosymmetric polymorph [12]. The crystalline KNaNbOF_5_ was first synthesized by Antokhina *et al.* [13]. However, the crystal structure was not verified at the time. The details of the atomistic structure in the α-phase were reported by Poeppelmeier *et al.* several years later [12]. The other phase, β-KNaNbOF_5_, is centrosymmetric [10,14]. This phase was introduced for the first time by Vasiliev *et al.* [10]. Experimentally, both polymorphic phases can be made from similar combinations of starting materials. The key difference comes from the different K:Na ratio in the synthetic route. The α-phase occurs when the ratio of K:Na is greater than 1:1, while a smaller ratio results in the β-phase. Although the α- and β-phases of KNaNbOF_5_ have the same stoichiometry, the same polar structure of the framework [NbOF_5_]^2−^, and very similar synthetic routes, they have very different properties.

In order to gain a comprehensive understanding of the physical properties of KNaNbOF_5_, detailed investigations are desirable. In this paper, employing the density functional theory (DFT), detailed computational studies of the electronic, mechanical, piezoelectric, and acoustic properties of KNaNbOF_5_ are carried out for both the α and β polymorphic phases. The property differences between the two phases are compared and discussed in detail.

## 2. Theoretical Method

The DFT calculations are performed with the Vienna Ab initio Simulation Package (VASP) [15]. The electron-ion interactions are represented by the projector augmented wave (PAW) method [16]. The electronic exchange correlation energy is treated using the generalized gradient approximation suggested by Perdew, Burke, and Ernzerhof (GGA-PBE) [17]. The wave functions are expanded on a plane wave basis with kinetic energy cutoff set to 600 eV. The Brillouin zone is sampled on a mesh of 6 × 10 × 8 *k*-points within the Monkhorst-Pack scheme [18] for α-KNaNbOF_5_, and a 10 × 10 × 8 *k*-point mesh for β-KNaNbOF_5_, respectively. The results of the total energy and the Hellmann-Feynman forces are convergent within 10^−4^ meV and 1 meV/Å, respectively.

It has been reported that the [NbOF_5_]^2−^ polar structure results in the special piezoelectric properties of KNaNbOF_5_ [12]. In order to describe this special structure, some parameters are used to describe the distorted octahedral [NbOF_5_]^2−^ [19]. The Baur’s distortion index *D* [20], based on bond lengths, is defined as:

(1) $$D=\frac{1}{n}\sum_{i=1}^{n}\frac{\left|l_{i}-l_{av}\right|}{l_{av}}$$

where *l_i_* is the distance from the central atom to the *i*th coordinating atom, and *l_av_* is the average bond length. The quadratic elongation, <λ> [21], is defined as:

(2) $$<\text{λ}>\;=\frac{1}{n}\sum_{i=1}^{n}{\left(\frac{l_{i}}{l_{0}}\right)}^{2}$$

where *l*_0_ is the center-to-vertex distance of a regular polyhedron of the same volume. <λ> is a dimensionless quantity that provides a quantitative measurement of the polyhedral distortion in a crystal, independent of the polyhedral size. The bond angle variance, σ*_2_* [21], is calculated by:

(3) $${\text{σ}}^{2}=\frac{1}{m-1}\sum_{i=1}^{m}{\left({\text{ϕ}}_{i}-{\text{ϕ}}_{0}\right)}^{2}$$

where *m* is (number of faces in the polyhedron) × 3/2, ϕ*_i_* is the *i*th bond angle, and ϕ_0_ is the ideal bond angle for a regular polyhedron.

## 3. Results and Discussion

The noncentrosymmetric α-KNaNbOF_5_ belongs to the orthorhombic system with the space group Pna2_1_, while the centrosymmetric β-KNaNbOF_5_ is a tetragonal system with a space group of P4/nmm. The lattice parameters in [10,12] are: *a* = 11.8653(11) Å, *b* = 5.8826(6) Å, *c* = 8.1258(8) Å for α-KNaNbOF_5_; *a* = 5.9352(2) Å, *c* = 8.5487(5) Å for β-KNaNbOF_5_, respectively. Starting from these experimental parameters, the structures in our calculations are obtained by minimizing the total energy.

### 3.1. Atomic Structure and Bonding Properties

Orthorhombic (α-) and tetragonal (β-) KNaNbOF_5_ are investigated both within the local density approximation (LDA) and the generalized gradient approximation (GGA-PBE). Our estimations of the lattice parameters are listed in Table 1, with LDA, PBE, and experimental results listed for comparison. It is well known that LDA lever calculations usually underestimate and GGA-lever calculations overestimate the lattice constants, which also occurs in our calculations. Since the PBE lattice constants of the β phase are closer to the experimental values (the error is around 1.47%–1.77%), the PBE function is used for further calculations.

**Table 1:** The calculated lattice constants and the unit-cell volume in α- and β-KNaNbOF_5_. Δ*a*, Δ*b* and Δ*c* are the relative errors of the calculated lattice constants compared to the experimental values. LDA = Local density approximation; PBE = Perdew, Burke, and Ernzerhof approximation; Exp. = Experimental values.

Phase	Method	*a* (Å)	*b* (Å)	*c* (Å)	Volume (Å^3^)	Δ*a* (%)	Δ*b* (%)	Δ*c* (%)
α-phase	Exp. [12]	11.865	5.883	8.126	567.171	-	-	-
	LDA	11.643	5.779	7.994	537.880	−1.87	−1.76	−1.63
	PBE	12.098	5.982	8.300	600.777	+1.96	+1.70	+2.15
β-phase	Exp. [10]	5.935	5.935	8.549	301.142	-	-	-
	LDA	5.816	5.816	8.385	283.629	−2.01	−2.01	−1.91
	PBE	6.040	6.040	8.674	316.502	+1.77	+1.77	+1.47

**Figure 1:**
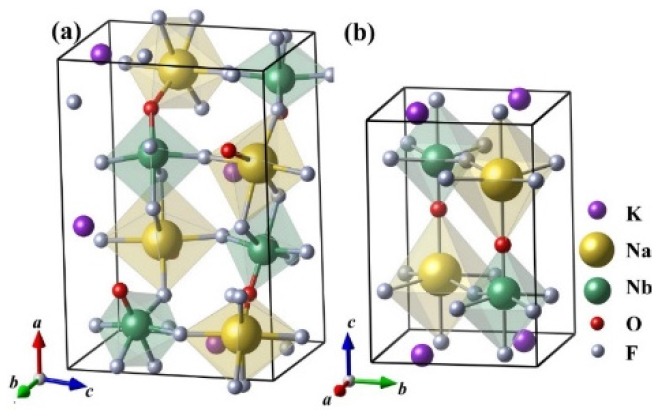
The crystal structures of KNaNbOF_5_ in different phases. The purple, golden, green, red and white spheres are K, Na, Nb, O and F atoms, respectively. The Na- and Nb-centered octahedra are also shown. (**a**) The orthorhombic noncentrosymmetric phase of KNaNbOF_5_ (α-phase, space group Pna2_1_); (**b**) The tetragonal centrosymmetric phase of KNaNbOF_5_ (β-phase, space group P4/nmm). The crystal axes are also shown for clarity.

It can be found that α- and β-KNaNbOF_5_ are very similar to each other, as shown in Figure 1. Both crystal structures consist of [NbOF_5_] and [NaOF_5_] octahedron sharing vertices to form three-dimensional networks, with the K atoms occupying the interstitial sites. In the framework of [NaOF_5_] and [NbOF_5_], the Na and Nb atoms are coordinated by five F and one O atoms in a distorted octahedral arrangement. There are also obvious differences between them in crystal structure. Firstly, in α-KNaNbOF_5_, the alternating NbOF_5_ octahedra share both vertices and F-F edges, while in β-KNaNbOF_5_, all the NbOF_5_ octahedra share only vertices. Secondly, the K atoms are coordinated by six F atoms and one O atom in α-KNaNbOF_5_, while the K atoms are coordinated by 8 F atoms in the β phase, without any surrounding O atoms in β-KNaNbOF_5_. Thirdly, in α-KNaNbOF_5_, all the atoms occupy the Wyckoff positions 4a, while in β-KNaNbOF_5_, the K atoms occupy the Wyckoff position 2a; the Na, Nb and O atoms occupy positions 2c; and the remaining inequivalent fluorides occupy two kinds of Wyckoff positions (2c and 8j).

In order to make a detailed analysis, the Baur’s distortion index *D*, the quadratic elongation <λ>, and the bond angle variance σ*^2^* can be used to evaluate the distortion of the structure, which are listed in Table 2. The effective charge for each atom (charge difference after bonding) is determined using Bader charge analysis [22,23], which is given in Table 3 with the corresponding calculated atomic positions. It can be found that the calculated values agree well with the experimental data. From these values, several conclusions can be made: (1) The Na-O and Na-F bond lengths are larger than that of Nb-O and Nb-F. Thus, the [NaOF_5_] occupies a larger volume than [NbOF_5_]; (2) The bond angle variances, σ*^2^*, are larger in [NaOF_5_] than [NbOF_5_] octahedra, both in α- and β-KNaNbOF_5_; (3) The Nb-O bond length in the α-phase (1.776 Å) is noticeably longer than that in the β-phase (1.757 Å); (4) The ionic formula of both α- and β-KNaNbOF_5_ can be defined as K^+0.90^Na^+0.90^[NbOF]_5_^−1.80^; (5) The effective charges on fluorides are different due to their different surroundings (−0.791–−0.698 for α-phase, and −0.813–−0.708 for β-phase).

**Figure 2:**
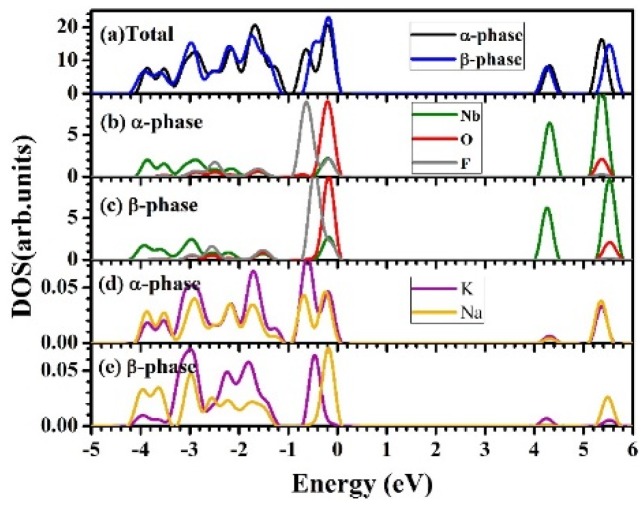
The calculated Density of States (DOS) of α- and β-KNaNbOF_5_ are plotted in (**a**), while the Projected Density of States (PDOSs) of Nb, O and F are plotted in (**b**,**c**). The negligible PDOSs of K and Na are also shown in (**d**,**e**) for comparison. The Fermi level is set to zero. The PDOSs of different atoms are plotted by the same colors as in Figure 1.

To analyze the electronic structure of KNaNbOF_5_, the electronic density of states (DOS), is illustrated in Figure 2. Although there are many differences in the structures between α- and β-KNaNbOF_5_, their density of states is similar to each other, indicating that their bonding characters are similar. The projected density of states (PDOS) of F, O and Nb are localized, indicating their ionic characteristics. Both α- and β-KNaNbOF_5_ are insulators with band gaps of about 4 eV. Although the band gap error is generally not definite due to the PBE approximation, the calculation method usually underestimates the value in most cases. Thus, it is very probable that the actual band gap is greater than 4 eV. Further experimental investigations might be necessary to confirm this result. From the PDOS, we find the peak near the Fermi surface (the top of the valence band) mainly consists of O-*2p*, F-*2p* and Nb-*4d* states. The −0.4 eV peak is mainly contributed by the F-*2p* state. Compared with the PDOS of F, O, and Nb, the PDOS of Na and K are negligible between −5 and 6 eV, as shown in Figure 2d,e. The bottom of the conduction band is mainly contributed by the Nb-*4d* states, which splits into two sub-bands. The peaks at 5.4 eV for the α-phase and at 5.5 eV for the β-phase should be the anti-bonding states contributed by O-*2p* and Nb-*4d*. This reflects a little covalent characteristic in the O-Nb interaction.

**Figure 3:**
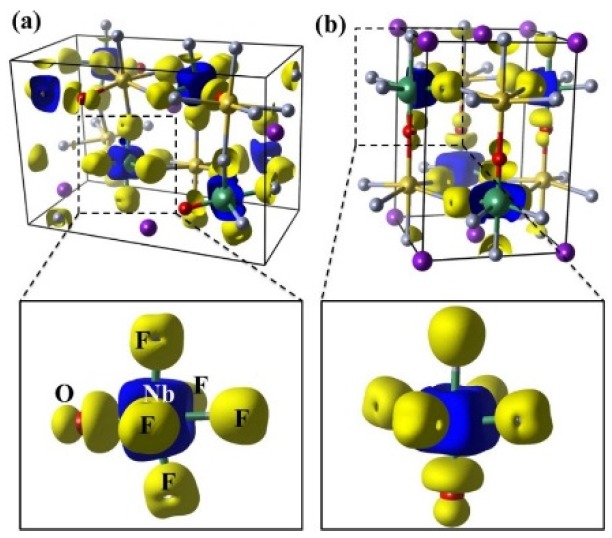
Deformation charge density (difference between the crystal charge and the atomic charge distribution) of KNaNbOF_5_ in (**a**) the α-phase; and (**b**) the β-phase. The yellow and blue isosurfaces (1.5 × 10^−2^ electrons/Bohr^3^) correspond to the electron increase and the depletion zone, respectively.

In order to further reveal the bonding properties of KNaNbOF_5_, the charge differences between the crystal charge and the atomic charge are depicted in Figure 3. The positive values mainly locate on the F and the O atoms, while the negative values mainly locate on the Nb atoms. This charge transfer indicates that the crystal is held together mainly by the ionic interactions between the Nb cations and the F/O anions. Moreover, the distribution of electrons gained by O-*2p* is obviously asymmetric. The electron charge densities on the O-Nb bond are higher, illustrating a little covalent bonding characteristic between the Nb and the O. All these results are consistent with our previous DOS analysis.

**Table 2:** The geometric properties of the [NbOF_5_] and [NaOF_5_] octahedra of α-NaNbOF_5_ and β-NaNbOF_5_, including the average bond length *l_av_* (in Å), the polyhedral volume *V* (in Å^3^), the distortion index *D*, the quadratic elongation *<*λ*>,* and the bond angle variance σ*^2^* (in degree^2^). The values calculated from experimental data in [10,12] are listed in brackets.

Phase	Octahedra	*l_av_*	*V*	*D*	<λ>	σ^2^
α-phase	[NbOF_5_]	1.985	10.215	0.036	1.017	48.784
		(1.942)	(9.592)	(0.034)	(1.016)	(44.311)
	[NaOF_5_]	2.342	16.700	0.020	1.018	58.982
		(2.300)	(15.786)	(0.027)	(1.019)	(63.760)
β-phase	[NbOF_5_]	1.990	10.230	0.039	1.023	65.121
		(1.944)	(9.546)	(0.040)	(1.022)	(63.543)
	[NaOF_5_]	2.337	16.663	0.010	1.015	51.677
		(2.310)	(16.118)	(0.011)	(1.014)	(48.468)

**Table 3:** The calculated atomic positions for KNaNbOF_5_ with Pna2_1_ symmetry for the α-phase and P4/nmm for the β-phase, respectively. The calculated lattice parameters are listed in Table 1. WP refers to Wyckoff position, MP refers to multiplicity, and *Q* refers to the atomic effective charge. The experimental data in [10,12] are listed in brackets.

Phase	Atom	WP	MP	*x*	*y*	*z*	*Q* (e)
α-phase	K	a	4	0.0398(0.0385)	0.4685(0.4680)	0.0955(0.0927)	0.903
	Na	a	4	0.1373(0.1389)	0.9509(0.9533)	0.3401(0.3414)	0.896
	Nb	a	4	0.8480(0.8492)	0.0407(0.0420)	0.3328(0.3334)	2.796
	O	a	4	0.7219(0.7225)	0.8998(0.9016)	0.2919(0.2940)	−0.977
	F1	a	4	0.0023(0.0029)	0.2167(0.2169)	0.3701(0.3700)	−0.791
	F2	a	4	0.8731(0.8725)	0.1485(0.1472)	0.1093(0.1090)	−0.702
	F3	a	4	0.9529(0.9527)	0.7849(0.7853)	0.2979(0.2976)	−0.720
	F4	a	4	0.8595(0.8609)	0.9625(0.9650)	0.5655(0.5650)	−0.708
	F5	a	4	0.7874(0.7879)	0.3353(0.3356)	0.3956(0.3956)	−0.698
β-phase	K	a	2	0.5000(0.5000)	0.5000(0.5000)	0.0000(0.0000)	0.903
	Na	c	2	0.0000(0.0000)	0.5000(0.5000)	0.2668(0.2660)	0.891
	Nb	c	2	0.0000(0.0000)	0.5000(0.5000)	0.7348(0.7351)	2.768
	O	c	2	0.0000(0.0000)	0.5000(0.5000)	0.5322(0.5351)	−0.915
	F1	c	2	0.0000(0.0000)	0.5000(0.5000)	0.9891(0.9865)	−0.813
	F2	j	8	0.2304(0.2295)	0.2696(0.2705)	0.7723(0.7719)	−0.708

### 3.2. Piezoelectricity and Acoustic Properties

The crystallographic symmetry of materials plays an important role in the piezoelectric phenomena. According to the definition of the piezoelectric effect, the piezoelectric tensor of α-KNaNbOF_5_ is:

(4) $$\left[\begin{array}{cccccc} 0 & 0 & 0 & 0 & e_{x5} & 0\\ 0 & 0 & 0 & e_{y4} & 0 & 0\\ e_{z1} & e_{z1} & e_{z3} & 0 & 0 & 0 \end{array}\right]$$

The calculated piezoelectric components of α-KNaNbOF_5_ are listed in Table 4, in which the data of ZnO are also listed. It is well known that ZnO has good piezoelectric properties, and has been widely used in filters for incoming television signals [24,25]. ZnO ceramics are also widely used as varistors for surge protection [26]. Compared with ZnO, the piezoelectric constants of α-KNaNbOF_5_ are much lower. In order to make a comparison to the experimental piezoelectric response measurement reported in [9], the calculated piezoelectric stress matrix [*e*] is converted into the piezoelectric strain matrix [*d*] by the relationship [*e*] = [*c*][*d*], where [*c*] is the elastic matrix. Based on Table 4 and Table 5, the calculated *d_z3_* is 1.5 pC/N, which is lower than the experimental value 6.7 pC/N [9]. Since our calculated lattice parameters, atomic positions, and relative permittivity agree well with experiments, the errors in the calculated *d_z3_* may originate from three aspects: (1) The experiment was carried out at room temperature—since finite temperatures are not included in the calculation, the calculated value of *d_z3_* is smaller than that observed in experiments; (2) The value of *d_z3_* is sensitive to the crystal structure—the calculated lattice constants deviate from the experimental values by ~1%, which affects the evaluation of *d_z3_*; (3) Compared to other commonly used piezoelectric materials, such as BaTiO_3_ (~190 pC/N) and K_0.5_Na_0.5_NbO_3_ (~160 pC/N) [9], the *d_z3_* value of KNaNbOF_5_ is much lower. Thus, although the relative error of the calculated *d_z3_* is large, the absolute error is only 4.8 pC/N. From these values, we can conclude that the piezoelectricity of α-KNaNbOF_5_ is not very strong. This is consistent with the fact that the piezoelectricity of α-KNaNbOF_5_ arises from the competition between primary and secondary distortions [11,12]. In the centrosymmetric structure of β-KNaNbOF_5_, the calculated piezoelectric components are all zero as expected from the symmetry of the crystal.

**Table 4:** The calculated piezoelectric tensor elements *e_ij_* of α-KNaNbOF_5_. The data of ZnO are listed for comparison. The piezoelectric components of β-KNaNbOF_5_ are all zero.

*e_ij_* (C/m^2^)	*e_x_*_5_	*e_y_*_4_	*e_z_*_1_	*e_z_*_2_	*e_z_*_3_
α-KNaNbOF_5_	0.11	0.12	−0.07	−0.08	0.05
ZnO [27]	-	−0.59	−0.61	1.14	-

**Table 5:** The calculated elastic constants of the two phases of KNaNbOF_5_ (in GPa).

Phase	*C*_11_	*C*_12_	*C*_13_	*C*_22_	*C*_23_	*C*_33_	*C*_44_	*C*_55_	*C*_66_
α-phase	50.9	26.8	13.8	51.2	19.7	64.2	11.0	17.1	20.2
β-phase	51.1	25.6	22.5	-	-	63.0	10.3	-	26.7

In experiments, the ultrasonic wave velocities of certain directions can be measured to obtain the elastic constant by the Christoffel equation [27,28]. The Christoffel equation for β-KNaNbOF_5_ is written as:

(5) $$\left(l_{iK}C_{KL}l_{Lj}-{\text{δ}}_{jk}\text{ρ}V^{2})({\text{α}}_{j})=0\;(j,\;k=1,2,3\right)$$

where *C_KL_* is the elastic constant (from Table 5), *l* is the propagation matrix, δ*_jk_* is the Kronercker sign, ϱ is the density of crystal, α*_j_* is the eigenvector, and *V* is the velocity of acoustic wave.

Since α-KNaNbOF_5_ has piezoelectric property, the quasistatic approximation is used to transform the conventional Christoffel equation to the stiffened Christoffel equation. In this approximation, the effect of the quasistatic electric field is saved. The stiffened Christoffel equation is written as:

(6) $$k^{2}\left\{l_{iK}\left[C_{KL}+\frac{\left(e_{Kj}l_{j})(l_{i}e_{iL}\right)}{l_{i}\left({\text{ε}}_{ij}-e_{iI}S_{IJ}e_{Ji}^{\prime }\right)l_{j}}\right]l_{Lj}\right\}{\text{α}}_{j}=\text{ρ}{\text{ω}}^{2}{\text{α}}_{i}$$

where *C_KL_* is the elastic constant (from Table 5), *S_IJ_* corresponds to the components of the elastic compliance matrix, ϱ is the density of crystal (from Table 1), ω is the phase velocity of the acoustic wave, α is the eigenvector, *e* is the piezoelectric stress tensor (from Table 4), ε is the dielectric constant (from Table 6), and *l* is the propagation matrix. All of these values are obtained from the first-principles calculations.

**Table 6:** Values of electronic and ionic contributions and total value of the relative dielectric permittivity (ε*_ij_*) for α- and β-KNaNbOF_5_.

Phase	ε*_ij_*	Electronic	Ionic	Total
α-phase	ε*_xx_*	4.05	2.24	6.29
	ε*_yy_*	3.70	2.16	5.86
	ε*_zz_*	3.68	2.13	5.81
β-phase	ε*_xx_*	4.74	2.03	6.77
	ε*_yy_*	4.74	2.03	6.77
	ε*_zz_*	2.51	2.19	4.70

The elastic stiffness (*C_KL_*) defines the resistance of the material to undergo strain under the action of a mechanical stress within the elastic regime. The orthorhombic (α-KNaNbOF_5_) and the tetragonal crystal (β-KNaNbOF_5_) have nine and six independent elastic constants, respectively. The calculated elastic constants are listed in Table 5. The relative dielectric permittivities for α-KNaNbOF_5_ are ε*_xx_* = 6.29, ε*_yy_* = 5.86 and ε*_zz_* = 5.81. For the β phase, they are ε*_xx_* = ε*_yy_* = 6.77 and ε*_zz_* = 4.70, as shown in Table 6. These calculated results are close to the experimental measured relative permittivity (ε = 7.1) for α-KNaNbOF_5_ [9].

**Figure 4:**
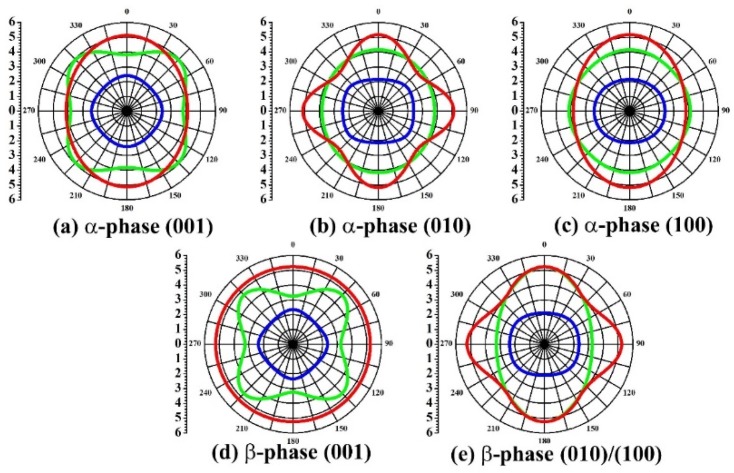
Slowness curves of acoustic waves for α- and β-KNaNbOF_5_. The slowness curves of α-KNaNbOF_5_ in (001), (010) and (100) planes are (**a**), (**b**) and (**c**), while those of β-KNaNbOF_5_ in (001) and (010)/(100) planes are (**d**) and (**e**), respectively. The inner blue circle represents the quasi-longitudinal wave and the middle green and outer red circles represent the other two quasi-transverse waves. The units of the slowness values are 10^−4^ s/m.

For a given direction, three velocities are determined by solving the Christoffel and the stiffened Christoffel equation. By changing the propagation direction, we obtain the velocity as a function of propagation direction. Usually, in practice, it is more convenient to use the slowness surface (the inverse of the velocity). We calculate the slowness curves of KNaNbOF_5_ for both the α- and β-phases and illustrate them in Figure 4 (for the tetragonal phase, the (010) plane is the same as the (001) plane). It is clear that there are three acoustic waves in each direction: one quasi-longitudinal mode and two quasi-transverse modes. The velocities of quasi-longitudinal modes are much larger than those of the other two in both phases of KNaNbOF_5_. These acoustic properties of KNaNbOF_5_ significantly change when a phase transition occurs: (1) For α-KNaNbOF_5_, the largest velocity is the quasi-longitudinal mode along the (001) direction, indicating that the velocity reaches maximum along this direction, while for β-KNaNbOF_5_, the velocity is at a maximum along (110) and (001); (2) For α-KNaNbOF_5_, the maximum values of the slowness surface are along (100) and (001), while for β-KNaNbOF_5_, all the directions in the (001) plane have the same maximum values of slowness; (3) All the acoustic waves of α-KNaNbOF_5_ are anisotropic, however, in the (001) plane of β-KNaNbOF_5_, the propagation of the outer acoustic wave is obviously isotropic. This mode is a pure transverse mode, in which the polarization is normal to the direction of propagation. (4) The slowness curves are very different on the (100) plane between α- and β-phases.

### 3.3. Mechanical Properties

From the elastic constants (*C_KL_*) listed in Table 5, more elastic-relevant properties can be obtained. The mechanical stability of KNaNbOF_5_ can be determined by the Born-Huang criterion [29]. For the α-phase, they are:

(7) $$\begin{array}{l} C_{11}>0,C_{22}>0,C_{33}>0,\\ C_{44}>0,C_{55}>0,C_{66}>0,\\ {}[C_{11}+C_{22}+C_{33}+2(C_{12}+C_{13}+C_{23})]>0,\\ (C_{11}+C_{22}-2C_{12})>0,\\ (C_{11}+C_{33}-2C_{13})>0,\\ (C_{22}+C_{33}-2C_{23})>0 \end{array}$$

And for the β-phase, they are:

(8) $$\begin{array}{l} C_{11}>0,C_{33}>0,C_{44}>0,C_{66}>0,\\ C_{11}-C_{12}>0,\\ C_{11}+C_{33}-2C_{13}>0,\\ 2(C_{11}+C_{12})+C_{33}+4C_{13}>0 \end{array}$$

The calculated elastic constants listed in Table 5 satisfy the mechanical stability criterion. Using the calculated elastic constants, we further calculate the bulk modulus, shear modulus, Young’s modulus, and Poisson ratio to obtain a complete description of the mechanical behavior. The Voigt and the Reuss approximations [30] are used to estimate the bulk modulus and the shear modulus.

Since α-KNaNbOF_5_ belongs to the orthorhombic structure, the bulk and the shear moduli (*B* and *G*) for orthorhombic structures can be obtained from the general expression of the bulk and the shear modulus in Voigt-Reuss-Hill approximation. For the Voigt approximation, they are:

(9) $$B_{V}=[(C_{11}+C_{22}+C_{33})+2(C_{12}+C_{13}+C_{23})]/9$$

(10) $$G_{V}=[(C_{11}+C_{22}+C_{33})-(C_{12}+C_{13}+C_{23})+3(C_{44}+C_{55}+C_{66})]/15$$

While by the Reuss approximation, they are:

(11) $$\begin{array}{l} B_{R}=\Delta /[C_{11}(C_{22}+C_{33}-2C_{23})+C_{22}(C_{33}-2C_{13})-2C_{33}C_{12}\\ +C_{12}(2C_{23}-C_{12})+C_{13}(2C_{12}-C_{13})+C_{23}(2C_{13}-C_{23})] \end{array}$$

(12) $$\begin{array}{l} G_{R}=15/\{4[C_{11}(C_{22}+C_{33}+C_{23})+C_{22}(C_{33}+C_{13})+C_{33}C_{12}\\ -C_{12}(C_{23}+C_{12})-C_{13}(C_{12}+C_{13})-C_{23}(C_{13}+C_{23})]/\Delta \\ +3(1/C_{44}+1/C_{55}+1/C_{66})\} \end{array}$$

(13) $$\Delta =C_{13}(C_{12}C_{23}-C_{13}C_{22})+C_{23}(C_{12}C_{13}-C_{23}C_{11})+C_{33}(C_{11}C_{22}-C_{12}^{2})$$

where *B* and *G* are the bulk and the shear moduli obtained by Voigt or Reuss approximations, respectively (with subscripts *_V_* or *_R_*).

The β-KNaNbOF_5_ belongs to the tetragonal structure. Therefore, the formulas become:

(14) $$B_{V}=[(C_{11}+C_{22}+C_{33})+2(C_{12}+C_{13}+C_{23})]/9$$

(15) $$G_{V}=[(C_{11}+C_{22}+C_{33})-(C_{12}+C_{13}+C_{23})+3(C_{44}+C_{55}+C_{66})]/15$$

(16) $$\begin{array}{l} B_{R}=\Delta /[C_{11}(C_{22}+C_{33}-2C_{23})+C_{22}(C_{33}-2C_{13})-2C_{33}C_{12}\\ +C_{12}(2C_{23}-C_{12})+C_{13}(2C_{12}-C_{13})+C_{23}(2C_{13}-C_{23})] \end{array}$$

(17) $$\begin{array}{l} G_{R}=15/\{4[C_{11}(C_{22}+C_{33}+C_{23})+C_{22}(C_{33}+C_{13})+C_{33}C_{12}\\ -C_{12}(C_{23}+C_{12})-C_{13}(C_{12}+C_{13})-C_{23}(C_{13}+C_{23})]/\Delta \\ +3(1/C_{44}+1/C_{55}+1/C_{66})\} \end{array}$$

(18) $$\Delta =C_{13}(C_{12}C_{23}-C_{13}C_{22})+C_{23}(C_{12}C_{13}-C_{23}C_{11})+C_{33}(C_{11}C_{22}-C_{12}^{2})$$

Within the Voigt-Reuss-Hill approximation [30], the bulk modulus *B* and the shear modulus *G* are the average of the values by Voigt and Reuss approximations. The Young’s modulus (*E*)*,* the Poisson ratio (*v*), the velocity of the transverse (*V_t_*), the longitudinal (*V_l_*) acoustic wave, the average velocity (*V_a_*), and the Debye temperature (Θ*_D_*) can be obtained by:

(19) E=9BG/(3B+G),v=(3B−2G)/(6B+2G)

(20) $$V_{l}=\sqrt{(3B+4G)/3\text{ρ}},V_{t}=\sqrt{G/\text{ρ}},V_{a}={\left[\frac{1}{3}(\frac{2}{V_{t}^{3}}+\frac{1}{V_{l}^{3}})\right]}^{-1/3}$$

(21) $$\Theta_{D}=\frac{h}{k_{B}}{\left[\frac{3n}{4\pi }(\frac{N_{A}\text{ρ}}{M})\right]}^{1/3}V_{a}$$

where *h* is the Planck constant, *k_B_* is the Boltzmann constant, *n* is the number of atoms in the formula unit, *N_A_* is the Avogadro number, ϱ is the density of the crystal, and *M* is the molecular weight. The constants describing the mechanical behavior of KNaNbOF_5_ in the elastic regime are given in Table 7.

The bulk modulus *B* of a material determines the resistance to compression under a given hydrostatic pressure [31]. The shear modulus *G* describes the resistance of a material to deform under a shear stress. The calculated bulk modulus of KNaNbOF_5_ is only 31.9–34.0 GPa, which is much lower than steel (approximately 160 GPa) and comparable to glass (35–55 GPa) [32]. Therefore, neither α- nor β-KNaNbOF_5_ are hard materials. The bulk modulus *B* is twice the shear modulus, indicating that the parameter limiting the material’s stability is the shear modulus.

For the application of KNaNbOF_5_, the brittle or ductile behavior is of great importance. The ratio of bulk modulus to shear modulus (*B/G*) is frequently used to discriminate the brittle properties of materials [33]. According to the criterion given by Pugh [34], a material is brittle if the *B/G* ratio is less than 1.75. Otherwise, it behaves in a ductile manner. In the case of KNaNbOF_5_, the B/G ratio is 1.99–2.31, indicating that KNaNbOF_5_ is predominantly ductile. Since the B/G ratio of the β-phase is larger than that of the α-phase, we predict that β-KNaNbOF_5_ is more ductile.

**Table 7:** The calculated elasticity-relevant properties of three different phases of KNaNbOF_5_. The bulk modulus *B* and the shear modulus *G* within the Voigt and Reuss approximation (with subscripts *V* and *R*, respectively) are listed. Based on *B* and *G*, the Young’s modulus *E*, the Poisson ration *v*, the acoustic wave velocities (*V_l_*, *V_t_*, *V_a_*), and the Debye temperature Θ*_D_* are calculated (referring to Equations (9)–(21)).

Property	α-Phase	β-Phase
*B_V_* GPa)	31.9	34.0
*B_R_* (GPa)	31.8	33.8
*B* (GPa)	31.8	33.9
*G_V_* (GPa)	16.7	15.7
*G_R_* (GPa)	15.4	13.7
*G* (GPa)	16.0	14.7
*E* (GPa)	41.2	38.6
*v*	0.284	0.310
*V_l_* (m/s)	4253.1	4381.4
*V_t_* (m/s)	2335.5	2297.3
*V_a_* (m/s)	2603.3	2569.3
Θ*_D_* (K)	303.3	294.2

At low temperatures, the vibrational excitations arise solely from the acoustic modes of the phonon spectrum. Hence, the Debye temperature calculated using the elastic constants can be compared to the experimentally measured values. From Table 7, we find that the α-phase has a higher velocity of the longitudinal acoustic wave and a lower velocity of the transverse acoustic wave compared to the β-phase. The calculated velocity of the longitudinal acoustic wave is 4253–4381 m/s and the transverse acoustic wave is 2297–2336 m/s. The calculated velocities using the classic Debye model compare well with the velocities calculated from the Christoffel equation discussed above. The Debye temperature from the calculated acoustic velocities is 303 K for α-KNaNbOF_5_ and 294 K for β-KNaNbOF_5_. If the Debye temperature is low, the thermal conductivity is also low for insulators in general [31]. Heat is transported by two mechanisms in solids: lattice vibrations (phonons) and free electrons. Since KNaNbOF_5_ is an insulator, the scattering becomes quite large above the Debye temperature, making the solid a poor thermal conductor. The Debye temperature of KNaNbOF_5_ (294–303 K) is much lower than that of diamond (approximately 2230 K), indicating a poor thermal conductivity of the material.

## 4. Conclusions

In this work, we used density functional theory to study the electronic, piezoelectric, mechanical, and acoustic properties of the recently synthesized KNaNbOF_5_ crystal. The bonding and mechanical properties of α- and β-KNaNbOF_5_ are very similar. KNaNbOF_5_ is found to present a strong ionic characteristic with weak covalent bonding. The ionic formula can be defined as K^+0.90^Na^+0.90^[NbOF]_5_^−1.80^. Both α- and β-KNaNbOF_5_ are insulators with an energy band gap of about 4 eV. The top of the valence band mainly consists of O-*2p*, F-*2p* and Nb-*4d* states, while the bottom of the conduction band is mainly contributed by the Nb-*4d* states, which split into two sub-bands. The *B/G* ratio of KNaNbOF_5_ is 1.99–2.31, which is higher than the critical value of 1.75, indicating that it is predominantly ductile. The calculated bulk modulus of KNaNbOF_5_ is only 31.8–33.9 GPa, which is comparable to glass. In contrast, the calculated piezoelectric and acoustic properties of the two phases are very different. The small values of the piezoelectric components of α-KNaNbOF_5_ illustrate its poor piezoelectricity compared with ZnO, while the values of β-KNaNbOF_5_ are all zero, as expected from the symmetry of the crystal. Based on the quasistatic approximation, the slowness surface of the acoustic waves is calculated so as to describe its acoustic properties. A pure transverse mode is found in the (001) plane of β-KNaNbOF_5_, in which the propagation of the outer acoustic wave is obviously isotropic. These calculated results are an initial step towards characterizing the properties of KNaNbOF_5_.
